# The Effect of Combined Elicitation with Light and Temperature on the Chlorogenic Acid Content, Total Phenolic Content and Antioxidant Activity of *Berula erecta* in Tissue Culture

**DOI:** 10.3390/plants13111463

**Published:** 2024-05-25

**Authors:** Jan Senekovič, Terezija Ciringer, Jana Ambrožič-Dolinšek, Maša Islamčević Razboršek

**Affiliations:** 1 Faculty of Agriculture and Life Sciences, University of Maribor, Pivola 10, 2311 Hoče, Slovenia; jana.ambrozic@um.si; 2 Faculty of Natural Sciences and Mathematics, University of Maribor, Koroška Cesta 160, 2000 Maribor, Slovenia; terezija.ciringer@um.si; 3 Faculty of Education, University of Maribor, Koroška Cesta 160, 2000 Maribor, Slovenia; 4 Faculty of Chemistry and Chemical Engineering, University of Maribor, Smetanova Ulica 17, 2000 Maribor, Slovenia

**Keywords:** *Berula erecta*, Apiaceae, plant tissue culture, elicitation, phenolic compounds, chlorogenic acid, antioxidant activity

## Abstract

Chlorogenic acid is one of the most prominent bioactive phenolic acids with great pharmacological, cosmetic and nutritional value. The potential of *Berula erecta* in tissue culture was investigated for the production of chlorogenic acid and its elicitation combined with light of different wavelengths and low temperature. The content of chlorogenic acid in the samples was determined by HPLC-UV, while the content of total phenolic compounds and the antioxidant activity of their ethanol extracts were evaluated spectrophotometrically. The highest fresh and dry biomasses were obtained in plants grown at 23 °C. This is the first study in which chlorogenic acid has been identified and quantified in *Berula erecta*. The highest chlorogenic acid content was 4.049 mg/g DW. It was determined in a culture grown for 28 days after the beginning of the experiment at 12 °C and under blue light. The latter also contained the highest content of total phenolic compounds, and its extracts showed the highest antioxidant activity. *Berula erecta* could, potentially, be suitable for the in vitro production of chlorogenic acid, although many other studies should be conducted before implementation on an industrial scale.

## 1. Introduction

Phenolic acids are one of the most significant groups of bioactive compounds present in various plant sources [1]. Phenolic acids are divided into two major groups, hydroxybenzoic acids and hydroxycinnamic acids, which are derived from non-phenolic molecules of benzoic acid and cinnamic acid [2]. 5-caffeoylquinic acid, also well known as chlorogenic acid (and referred to further in the text as “chlorogenic acid”) [3], is a major product of phenylpropanoid metabolism [4] and is one of the hydroxycinnamic acid conjugates [5]. It is the most abundant isomer among caffeoylquinic acid isomers and one of the most available phenolic acids in plants [6]. Chlorogenic acid has attracted attention due to its anticancer, antioxidant and antibacterial activity [7]. Furthermore, it has various biological functions, including cardiovascular, nerve, kidney and liver protection [8]. Chlorogenic acid exhibits more significant antioxidant activity in vivo than many flavonoids [9], and it is characterised by relatively low toxicity and side effects [10]. Chlorogenic acid is recognised as an antioxidant, with growing interest in pharmacological, cosmetic and food applications [11]. 

The synthesis and subsequent accumulation of phenolic acids in plants occur mainly in response to environmental stresses. Consequently, the application of elicitors mimicking stress-associated effects can be applied to achieve in vitro high yields of secondary metabolites [12]. The combined application of different elicitors showed improved accumulation of secondary metabolites due to their synergistic effect [13]. Light is an effective abiotic elicitor that affects plant growth and development and regulates primary and secondary metabolisms [14]. It also promotes the biosynthesis of phenolic compounds [15]. Light intensity is directly proportional to the accumulation of natural antioxidants in in vitro cultures of various plants [16]. Changes in light wavelengths can shift the primary and secondary metabolism in plants, alter the plant’s metabolism and affect the accumulation of the plant’s functional products [17]. The enzymatic activities of phenylalanine ammonia-lyase, chalcone synthase, chalcone isomerase and flavonol synthase are integral components of the phenylpropanoid pathway, which leads to the biosynthesis of flavonoids and is observed to be stimulated either directly or indirectly in response to various light conditions [18].

Cold acclimation affects mainly carbohydrate metabolism, which results in the accumulation of soluble sugars, sugar alcohols, organic acids, amino acids, polyamines and substrates for secondary metabolism. It also affects the synthesis of plant cell wall compounds such as lignin or suberin, which is associated with the promotion of chlorogenic acid synthesis [19,20].

*Berula erecta (Huds.) Coville* is a fast-growing species that occurs in various freshwater habitats and is a sub-cosmopolitan species distributed in the Northern Hemisphere [21]. *Berula erecta* is also a fast clonally growing species, in a tissue culture system, on a simple growth medium, without growth regulators. Therefore, *Berula erecta* is a suitable plant species for cultivation in tissue culture [22]. Members of the Apiaceae family are a well-known source of natural antioxidants such as phenolic acids and flavonoids [23]. The extract of *Berula angustifolia* (a synonym for *Berula erecta* [24]) collected from the wild was rich in total phenolic compounds and showed high antioxidant activity [25]. As far as we know, no study has yet been conducted to determine chlorogenic acid in *Berula erecta*. Therefore, one of the main objectives of this research was to identify and quantify chlorogenic acid in *Berula erecta* grown in tissue culture. In our experimental system, we studied the in vitro cultivation of *Berula erecta* on solid Murashige and Skoog medium (MS) without growth regulators [26]. The industry uses in vitro culture systems for mass production of the bioactive compounds of medicinal plants to ensure a continuous supply in a relatively short period of time [27]. Although the in vitro production of phenolic compounds is well understood, there is still a lack of knowledge about the biosynthesis of individual phenolic compounds due to the complexity of the biosynthetic pathways and extraction procedures [28].

The aim of this study was to investigate whether *Berula erecta* can be a source of natural antioxidants such as chlorogenic acid. The main goal of the present study was to evaluate the total phenolic content, with emphasis on the particularly useful chlorogenic acid and the antioxidant activity of the ethanolic extract of *Berula erecta*. In addition, the combined elicitation of these substances was investigated with different light wavelengths and low-temperature conditions. The combined elicitation was tested because the chlorogenic acid content of *Berula erecta* had not been quantified until this study, and the aim was to stimulate chlorogenic acid biosynthesis as much as possible in order to evaluate the suitability of this species for cultivation to obtain chlorogenic acid based on the highest possible chlorogenic acid content.

## 2. Results

### 2.1. Influence of Light Quality and Temperature on Plant Development

**Figure 1:**
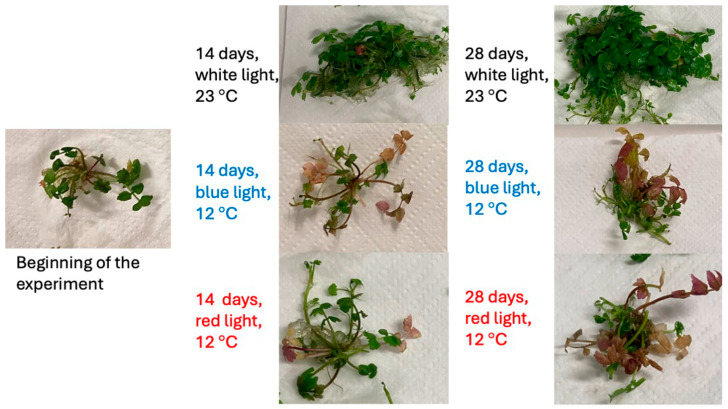
Morphologically visible changes in the tissue culture of *Berula erecta* grown under different light and temperature conditions.

Morphologically noticeable changes in plants grown for different periods of time under different light and temperature conditions are shown in Figure 1. Before the plants were exposed to the different treatments of combined elicitation, all the plants included in the experiment were the same size. Their leaves were bright green without visible chlorosis and necrosis. According to the morphologically observable changes, the plants grown at 23 °C under white light grew best. After 14 days, the plants were visibly larger and had the same colour as at the beginning of the experiment. After 28 days of the experiment, the plants were even larger than after 14 days of the experiment. Their colour had not changed either. There was no visible chlorosis or necrosis. Visible morphological changes occurred in plants grown under different light and temperature conditions. After 14 days, plants grown under blue light at 12 °C were approximately the same size as at the beginning of the experiment. A smaller number of leaves turned to purple shades. Some necrosis appeared. After 28 days from the beginning of the experiment, the plants were still about the same size as at the start of the experiment. Compared with the situation 14 days ago there were more purple-coloured leaves and less necrosis. Similar changes were observed in plants grown under red light at 12 °C. The difference between these two combined elicitations was shown 28 days after the experiment, when the plants grown under red light were slightly larger than the plants grown under blue light.

### 2.2. Influence of Light Quality and Temperature on Fresh and Dry Weight

**Figure 2:**
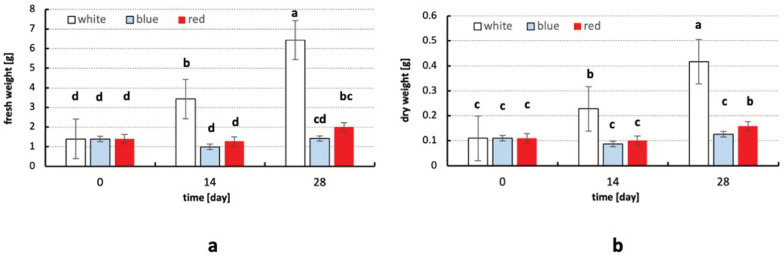
The effect of different light and temperature conditions on the average (**a**) fresh weight and (**b**) dry weight of *Berula erecta* in vitro after 14 and 28 days of the experiment. Means ± SD (n = 24) are shown. Significant differences are indicated by different letters (Kruskal–Wallis test, *p* < 0.05).

The best plant growth was observed under white light at 23 °C. Both fresh and dry weights increased during all 28 days of the experiment (Figure 1 and Figure 2) in the growth chamber (23 °C, white light), almost doubling after 14 days, and more than tripling after 28 test days.

A different trend in the change in fresh and dry mass was observed in plants exposed to lower-temperature conditions (12 °C) and different lighting conditions after 14 and 28 days of the experiment. The change in temperature and light wavelength slowed drastically, and almost halted, the growth and decreased both fresh and dry weights after 14 and 28 days of the experiment compared to the plants grown in the growth chamber at 23 °C (white light).

The fresh and dry weights of plants cultivated under blue and red lights and at a temperature of 12 °C decreased slightly, but not significantly, after 14 days of the experiment and increased after 28 days of the experiment. This increase was not significant under blue light conditions and was significant under red light conditions.

### 2.3. Influence of Light Quality and Temperature on Chlorogenic Acid Contents

The chlorogenic acid content differed significantly between all the experimental systems. The concentration range of the chlorogenic acid in *Berula erecta* grown in tissue culture under different temperature and light conditions was between 0.927 mg and 4.049 mg per gram of freeze-dried plant material. The biosynthesis of chlorogenic acid was most efficient under the high-energy blue light. Accordingly, the highest chlorogenic acid content was observed under blue light at 12 °C. The chlorogenic acid content increased on all 28 days of the experiment (Table 1) under blue light. After 14 days it was about 1.3 times higher, and, after 28 days, 2.1 times higher than at the beginning of the experiment.

A different trend in the changes in chlorogenic acid content was observed in plants exposed to different light conditions after 14 and 28 days of the experiment. Under white and red light, the chlorogenic acid content decreased after 14 days of the experiment but increased after 28 days of the experiment compared to the chlorogenic acid content at the beginning of the experiment.

### 2.4. Influence of Light Wavelength and Temperature on Total Phenolic Content

The total phenolic content (TPC) was significantly different in all the experimental systems. The highest TPC was observed under blue light at 12 °C. The concentration range of TPC in *Berula erecta* grown in tissue culture under different temperature and light conditions was between 14.76 mg of gallic acid and 28.12 mg of gallic acid per gram of freeze-dried plant material. The TPC increased on all 28 test days (Table 1) under blue light. After 14 days it was about 1.2 times higher and after 28 days about 1.6 times higher than at the beginning of the experiment.

A different trend in TPC changes was observed in plants exposed to different light conditions after 14 and 28 days of the experiment. Under white and red light, TPC decreased after 14 days of the experiment but increased after 28 days of the experiment compared to the TPC at the beginning of the experiment.

### 2.5. Influence of Light Wavelength and Temperature on the Antioxidant Activity of Ethanolic Extracts of Berula erecta

The highest antioxidant activity was observed in the extracts of plants grown under blue light at 12 °C. The antioxidant activity of the ethanolic extracts ranged from 0.0296 mmol of Trolox to 0.1008 mmol of Trolox per gram of freeze-dried plant material. The antioxidant activity increased during all 28 days of the experiment (Table 2) under blue light. It was approximately 1.3 times higher after 14 days and approximately 2 times higher after 28 days than at the beginning of the experiment.

A different trend of antioxidant activity in the extracts was observed in the plants exposed to different light conditions after 14 and 28 days of the experiment. Under white and red light, the antioxidant activity decreased after 14 days of the experiment but increased after 28 days of the experiment compared to the antioxidant activity of the extracts from plants sampled at the beginning of the experiment.

### 2.6. Correlations between TPC and Antioxidant Activity in Relation to Chlorogenic Acid Content

Pearson’s correlation coefficient was calculated to determine the changes in TPC and antioxidant activity in relation to chlorogenic acid content (Table 3). A high positive correlation was found between the chlorogenic acid content and the TPC (0.980 **). Similarly, a high positive correlation was found between the chlorogenic acid content and antioxidant activity of ethanolic extracts determined by a DPPH test (0.963 **).

### 2.7. Influence of Light Wavelength and Temperature on the Yield of Chlorogenic Acid in Tissue Culture of Berula erecta Grown In Vitro

The yield of chlorogenic acid per 1 L of MS medium obtained from a *Berula erecta* tissue culture exposed to combined elicitation was calculated based on data of the dry weight and its chlorogenic acid content. The yield of chlorogenic acid was significantly different in all the experimental systems. The highest yield of chlorogenic acid was observed under blue light at 12 °C. The mean yield of chlorogenic acid in *Berula erecta* grown in tissue culture under different temperature and light conditions ranged from 9.402 mg to 168.873 mg of chlorogenic acid per 1 L of MS medium. The yield of chlorogenic acid increased during all 28 days of the experiment (Table 4) under blue light. After 14 days it was about 1.1 times higher and after 28 days approximately 8.1 times higher than at the beginning of the experiment. The yield of chlorogenic acid increased statistically significantly in the plants grown under white light throughout the experiment. However, the trend was different for plants grown under red light. Under red light, the chlorogenic acid yield decreased after 14 days of the experiment but increased after 28 days of the experiment compared to the yield of chlorogenic acid of plants sampled at the beginning of the experiment.

## 3. Discussion

### 3.1. The Influence of Light Quality and Temperature on Plant Development

Based on the visible morphological changes, we can conclude that the best-developed plants were grown under white light at 23 °C. They grew successfully throughout the observation period (they were larger than in the previous observation). The colour of the leaves did not change, and neither chlorosis nor necrosis occurred. Chlorophyll is the pigment that gives plants their characteristic green colour [29]. Chlorophyll is a key biochemical component responsible for photosynthesis and is a physiological indicator of plant condition. Changes in chlorophyll content can be used to assess the impact of environmental stress [30], as the most common change in green plants under stress is the loss of chlorophyll [31]. Therefore, the plants grew visibly all the time under the mentioned treatment and their green colour did not change, so we assumed that they were not under stress and that the growth conditions were suitable. However, if we wanted to draw conclusions about the physiological response of chlorophyll in plants to these conditions, it would have to be quantified using different analytical techniques. 

The plants grown at 12 °C under blue or red light reacted similarly. After 14 days, the plants were approximately the same size as at the beginning of the experiment. In both cases, purple leaves appeared after 14 days, and after 28 days of the experiment there were even more. Necrosis also appeared after 14 days, but less so after 28 days of the trial. The only difference between the latter two treatments was that the plants grown under red light were slightly larger after 28 days compared to the plants grown under blue light. The appearance of purple leaves and necrosis was most likely related to exposure to a lower temperature, as the same changes were observed by Ambrožič-Dolinšek et al. [32] when they exposed *Berula erecta* to a temperature of 13 °C in order to investigate slow growth [32]. Leaf colour is determined by the ratio of chlorophylls, carotenoids and anthocyanins [33]. We assume that anthocyanins are responsible for the purple colouration of the leaves, as anthocyanins are the predominant sub-class of coloured flavonoids that consist of red, purple or blue pigmentations in various plants [34]. The colour intensity of red leaves is increased by light stress, cool temperatures and slight drought during the anthocyanin synthesis phase that precedes chlorophyll degradation [33]. The anthocyanins’ accumulation increased significantly in plant tissues in response to low-temperature stress [35]. In addition, the biosynthesis of anthocyanins in plant tissues responds to light quality, and they play a role in photoprotection [36]. However, the effects of different light qualities on anthocyanin accumulation vary, depending on the plant species [37]. In order to explain and understand the influence of combined elicitation with light of different wavelengths and low temperature on the accumulation of anthocyanins better, it would be necessary to identify and quantify the anthocyanins in plant tissue. In addition, the influence of light and temperature on their biosynthesis should be investigated separately. In the future, it would be appropriate to investigate the suitability of *Berula erecta* grown in tissue culture under combined elicitation for the production of anthocyanins. Anthocyanins, similar to chlorogenic acid, are useful as nutraceutical ingredients, and they offer numerous beneficial health effects [38]. It may be possible to obtain anthocyanins from the tissue culture of *Berula erecta* in addition to chlorogenic acid, which would represent an additional economic and operational advantage.

### 3.2. The Influence of Light Quality and Temperature on Fresh and Dry Weight of Plants

The conditions at 23 °C and under white light stimulated the production of fresh and dry biomass, while lower temperatures inhibited it. This was also confirmed by the work of Ambrožič-Dolinšek et al. [32]. In the aforementioned study, *Berula erecta* plants grown for two months at 23 °C gained more fresh and dry weight than plants grown at 13 °C [32]. In the present study, the highest fresh and dry biomasses were observed in plants grown for 28 days after the beginning of the experiment at 23 °C and under white light. Therefore, the optimal combination of temperature and light wavelength for the production of plant biomass in the tissue culture of *Berula erecta* is at 20–25 °C under white light.

The average fresh and dry biomass of the plants exposed to red or blue light did not increase compared to the plants exposed to white light. The lowest fresh and dry biomass content was observed in the plants grown for 14 days after the beginning of the experiment at 12 °C and under blue light. The second highest fresh and dry biomass content was observed in the plants grown for 14 days after the beginning of the experiment at 23 °C and under white light. The plants grown under red light achieved a higher average fresh and dry weight content after 14 and 28 days of the experiment than the plants grown under blue light. This was confirmed by the results of several other studies on different plant species, such as tomato seedlings, *Lactuca sativa* and *Oncidium* orchids [39,40,41,42,43], according to which, the lowest fresh weight was achieved in seedlings after exposure to blue light. This could be related to the fact that red light has a direct effect on the increase in plant biomass, while blue light has an indirect effect on the increase in plant biomass, as it accelerates photosynthetic processes [44].

Plants grown at lower temperatures generally show a lower level of growth and development [45]. This also applies to *Berula erecta*. Ambrožič-Dolinšek et al. [32] found that *Berula erecta* grows more slowly at 13 °C than at 23 °C. Nevertheless, in their experiment, plants grown under both treatments gained weight [32]. In the present study, plants exposed to light of different wavelengths were exposed simultaneously to a lower temperature. The almost stunted growth was therefore probably related to exposure to a mixture of lower temperature and different wavelengths. However, the effect of light of different wavelengths and low temperature on *Berula erecta* mass gain should be investigated separately in the future in order to describe the response of *Berula erecta* to them more accurately.

### 3.3. The Influence of Light Wavelength and Temperature on Chlorogenic Acid and Total Phenolics’ Content in Berula erecta

Temperature and light influenced the concentration of chlorogenic acid in plant material of *Berula erecta* cultivated in tissue culture. Chlorogenic acid was determined in all the ethanolic extracts from plants grown under all the tested conditions. This is also the first study in which chlorogenic acid in *Berula erecta* was determined and quantified. The plants cultivated for 28 days at 12 °C and under blue light contained the highest average content of chlorogenic acid. Considering that chlorogenic acid has many beneficial effects on health, such as cardioprotective effects, antitumour activity and neuroprotective effects [46], *Berula erecta* may be a potential source for the production of chlorogenic acid, especially when is elicited with combined elicitation with lower temperatures, such as 12 °C as in our experimental system, and blue light.

*Berula erecta* has the potential for in vitro production of chlorogenic acid. The chlorogenic acid content of *Berula erecta* was compared with the chlorogenic acid content of *Echinacea purpurea* cultivated in vitro in different types of large-scale reactors [47]. The concentration of biosynthesised chlorogenic acid was comparable to the present study in both observed reactors. In different types of reactors, the culture of *Echinacea purpurea* contained 4.4 to 4.9 mg chlorogenic acid per gram of plant DW [47]. According to the results of Wu et al. [47], the concentration of chlorogenic acid in *Berula erecta* is lower than, but still comparable to, the content of chlorogenic acid in the species *Echinacea purpurea* [47]. The potential of chlorogenic acid production in plant tissue cultures has been investigated in several other studies on different plant species [48,49,50,51,52]. The content of chlorogenic acid synthesised in the callus culture of *Varthemia persica* was lower (0.182 mg/g DW) than in the tissue culture *of Berula erecta* elicited with blue light and a lower temperature [48]. Different in vitro cultures of *Eryngium planum* L. all contained (0.013–0.066 mg/g DW) a lower content of chlorogenic acid than *Berula erecta* grown under all the conditions tested in the present study [49]. *Schisandra chinensis* culture cultivated on different mediums contained a maximum of 0.3843 mg/g, which is less than *Berula erecta* grown under any conditions tested in this study [50]. Leaf culture and callus culture of *Cynara cardunculus* also contained a lower concentration of chlorogenic acid (1.36 mg/g DW) compared to the tissue culture of *Berula erecta* cultivated under the combined elicitation conditions [51]. Nevertheless, *Cynara cardunculus* is described as a species suitable for the production of chlorogenic acid [53]. The range of chlorogenic acid contents in the plant tissue cultures of *Peucedanum japonicum* grown under different conditions was between 0.00 mg/g DW and 10.50 mg/g DW. The plant material which contained 10.50 mg/g DW was grown in plant tissue cultures for four months, which is a much longer period than was observed in the present study. In cultures of *Peucedanum japonicum* grown under all other tested conditions, the chlorogenic acid content was comparable to the chlorogenic acid content in *Berula erecta* [52].

*Berula erecta* grown in tissue culture elicited with a combination of low temperature (12 °C) and blue light contained quite a high concentration of chlorogenic acid and should be a suitable plant species for in vitro chlorogenic acid production. Therefore, the elicitation of chlorogenic acid biosynthesis in the plant tissue of *Berula erecta* should be investigated further. The production of chlorogenic acid in plant tissue cultures has many advantages, such as faster plant growth, independence from weather conditions and an aseptic environment [54]. Consequently, other scientists have studied the effect of chlorogenic acid elicitation in tissue cultures of different plant species. Further studies should investigate the influence of such elicitors on the biosynthesis of chlorogenic acid in *Berula erecta*. Elicitation with exposure to a mix of red and blue light [55], methyl jasmonate [56,57], salicylic acid [57], methyl salicylate [58], titanium (IV) ascorbates [58], zinc oxide nanoparticles [55] and the fungal elicitor *Phytophthora megasperma* [59] should be investigated in the future. It has also been reported that the production of secondary metabolites in plants can be stimulated by exposure to heavy metals [60]. In addition, *Berula erecta* has shown potential for phytoremediation of arsenic, lead and selenium in the past [21,22,61]. Therefore, it would be suitable to test the elicitation of chlorogenic acid in a tissue culture of *Berula erecta* with heavy metals in the future. In addition, it would be necessary to test the influence of elicitation with light of different wavelengths and exposure to low temperature separately. The limitation of the present study is that it determines the influence of combined elicitation (a combination of lower temperature and different wavelengths of light) on the biosynthesis of chlorogenic acid, while the individual effect of these two elicitors has not been determined yet. Research that would investigate the separate influence of light wavelength and temperature on the biosynthesis of chlorogenic acids in *Berula erecta* would provide an even more detailed insight into the physiological response of *Berula erecta* to light and temperature stress and thus enable a better optimisation of elicitation in the future.

**Figure 3:**
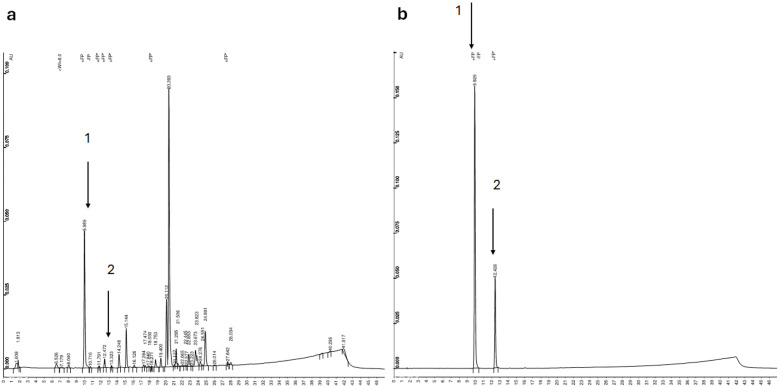
HPLC-UV profile (λ = 280 nm) of ethanolic extract from *Berula erecta* (**a**) and from the chlorogenic acid standard (**b**). 1. *trans*-chlorogenic acid and 2. *cis*-chlorogenic acid.

Chlorogenic acid has stronger antioxidant activity compared to many flavonoids [62]. However, we assume that *Berula erecta* also contains several other phenolic compounds in addition to chlorogenic acid according to the HPLC-UV chromatogram (Figure 3). In this study, only the effect of the combined elicitation on the biosynthesis of chlorogenic acid was investigated due to its broad application potential. However, many more studies are needed in the future to identify and quantify the phenolic compounds in *Berula erecta*. As far as we know, there are no studies examining phenolic acids in *Berula erecta*, and the present study is the first to identify and quantify chlorogenic acid in this plant species. Also, the flavonoids in *Berula erecta* are still relatively unexplored—according to the literature, it has been found to contain the flavonoid quercetin, mainly in the form of 3-glucoside and less in the form of 3-polyglycoside. In the aforementioned study, it was also found that *Berula erecta* does not contain kaempferol, isoharmentin, apigenin and luteolin [63]. Based on the purple-coloured leaves of plants grown under red and blue light at 12 °C, we assume that *Berula erecta* also contains anthocyanins. Therefore, it would be necessary to identify and quantify the complete phenolic profile of *Berula erecta* in further studies. Such a study would provide insight into what other useful phenolic compounds *Berula erecta* contains and if they can be co-produced efficiently with chlorogenic acid under the same combined elicitation. The co-production of metabolites has economic and operational advantages [64].

The TPC in *Berula erecta* varies similarly to the concentration of chlorogenic acid under different growing conditions. The only previous research that has investigated TPC in *Berula erecta* is the work of Tabaraki et al. [25]. These authors determined a total phenolic content of 23.08 mg GA/1 g DW in the water extract and 20.97 mg GA/1 g DW in the methanolic extract [25]. The levels of total phenolics determined in the study by Tabaraki et al. [25] were, therefore, within the range of the TPC values determined in this study. However, these concentrations are not comparable, as other extraction solvents were used in the study by Tabaraki et al. [25], and the plant material did not originate from a tissue culture. In the future, it would be useful to test several different solvents for the extraction of phenolic compounds from *Berula erecta* in order to determine the most effective one. In different plant species, the choice of extraction solvent has been shown to influence the amount of bioactive compounds extracted [65,66,67]. New trends in chemistry and in environmental legislation have placed demands on the use of so-called “green” extraction processes, so, in the future, it would also be suitable to test the efficiency of extraction with deep eutectic solvents [68].

It can be concluded that the most efficient conditions for the biosynthesis of phenolic compounds in *Berula erecta* were at 12 °C and under blue light. Kubica et al. [69] reported that blue light has a strong effect on the phenylpropanoid metabolic pathway, while red light has no direct effect on the biosynthesis of phenolic compounds [69]. The stimulatory effect of blue light on the activity of the phenylpropanoid metabolic pathway has been demonstrated in many previous studies. Cherry tomatoes cultivated under blue light contained the highest content of phenolic compounds [70]. The callus culture of *Operculina turpethum* also showed the highest concentration of phenolic compounds under blue light, followed by white and red light [71]. The content of chlorogenic acid in the in vitro culture of *Schisandra chinensis* changed in exactly the same way [72]. A similar change in the content of total phenolics and chlorogenic acid under different light conditions was also observed in *Berula erecta*.

Abiotic stress at low and high temperatures affects plant growth and metabolism [73]. In our experiment, the temperature was low (12 °C). The results suggest that light has a stronger effect on the biosynthesis of phenolic compounds than stress at low temperatures and that stress at low temperatures does not induce a significant response in the biosynthesis of phenolic compounds in the tissue culture of *Berula erecta*. The value of TPC and the content of chlorogenic acid decreased in plants exposed to low temperature and red light for 14 days, despite exposure to stress, while the content of both observed aforementioned parameters increased in plant material exposed to low temperature and blue light. However, after 28 days of acclimation, the TPC in plants cultivated at 12 °C and under red light was higher than 14 days after acclimation under the same conditions, but this increase of TPC after 28 days was observed in plant material which was cultivated under all treatments tested and could be the consequence of the consumption of some key nutrients from the culture medium and the resulting nutritional stress. Plants accumulate phenolic compounds under unfavourable conditions [74]. This was also confirmed by the results of the present study. In plants grown for 14 days after the beginning of the experiment at 23 °C and under white light (under the same conditions as the growth induction before), a decrease in TPC was observed, together with a decrease in the content of chlorogenic acid. This was probably due to the fact that the plants did not experience any change in temperature and wavelength of light and therefore did not experience any abiotic stress. The claim that nutritional stress occurred 28 days after the beginning of the experiment can be supported by tests on the plants that were still cultivated at 23 °C and under white light 28 days after the beginning of the experiment—we observed a significant increase in the TPC and also chlorogenic acid. These plants were not exposed to such a strong abiotic stress, but the content of chlorogenic acid and total phenolics increased. In different plant species, it was proved that nutritional stress of plants in tissue culture causes an increase in TPC [75,76,77].

### 3.4. The Influence of Light Wavelength and Temperature on the Antioxidant Activity of Plant Ethanolic Extracts of Berula erecta

Samples containing higher TPC and higher chlorogenic acid content also showed higher antioxidant activity of their extracts. Phenolic compounds can act as reducing agents, radical scavengers and suppressors of singlet oxygen formation [78]. A positive trend in the change in the concentration of phenolic compounds and antioxidant activity was also determined in many other studies. A simultaneous increase in phenolic compound content and antioxidant activity was found in rice [79], grape berry waste [80] and cherries [81]. The positive trend between the TPC and the antioxidant activity of the extracts also applied to *Berula erecta* in the present study. The reason for this is probably that environmental stress leads to increased biosynthesis of phenolic compounds in the plant and that phenolic compounds have antioxidant properties. In addition, plants respond to environmental stress with an increased ability to scavenge reactive oxygen species [82]. However, the antioxidant activity of ethanolic plant extracts is not only determined by phenolic compounds but also by other components, especially essential oils [83]. Lazarević, a Serbian researcher, and his colleagues found that (Z)-falcarinol is the most abundant compound in the essential oil of *Berula erecta* from Serbia [84]. The results of a 2017 study showed that falcarinol is a powerful antioxidant [85]. Ayla et al. [86] found that the most important compound in the essential oil of *Berula erecta* from Turkey is hexahydrofarnesylacetone [86]. Strong antioxidant activity has also been reported for hexahydrofarnesylacetone [87,88]. 

In the future, it would also be suitable to determine the antioxidant activity of the samples with other antioxidant activity assays, such as the FRAP assay and ABTS assay. The study of the antioxidant activity of the extracts of *Berula erecta* plants elicited with a combination of low temperature and different light wavelengths could also be extended by determining the content of ascorbic acid and the content of some endogenous enzymes—such as catalase, glutathione peroxidase and superoxide dismutase.

### 3.5. Correlations between TPC and Antioxidant Activity in Relation to Chlorogenic Acid Content

The positive correlations between TPC and antioxidant activity in relation to chlorogenic acid content were high. A high positive correlation between TPC and chlorogenic acid content was expected because chlorogenic acid has reactivity towards the Folin–Ciocalteu reagent [89], and in some studies it is even used as a standard instead of gallic acid in the method for determining TPC [90]. In previous studies, a high correlation between TPC and chlorogenic acid content was also determined in apples [91], coffee [92,93] and potatoes [94]. Based on this correlation, we can assume that the content of most phenolic compounds extracted from *Berula erecta* increases when exposed to the same combined elicitation in which the content of chlorogenic acid increases. In the future, however, it would be necessary to conduct studies that identify and quantify the other phenolic compounds in *Berula erecta*. It would also be necessary to evaluate the response of other main phenolics in *Berula erecta* to exposure to combined elicitation. Although phenolic compounds are often considered as a group of molecules with similar biological activity, the structure of polyphenols influences their activity and role in biological processes significantly, and, consequently, their involvement in plant stress responses [95]. 

A positive correlation between the antioxidant activity of the ethanolic extracts and the chlorogenic acid content was also expected, as chlorogenic acid has a strong DPPH-radical-scavenging activity [96,97]. This positive correlation suggests that other substances with antioxidant activity are also biosynthesised under the dual stimulation conditions that stimulate the biosynthesis of chlorogenic acid. In future studies, it would be useful to identify and quantify the antioxidant compounds in *Berula erecta*.

### 3.6. The Influence of Light Wavelength and Temperature on the Yield of Chlorogenic Acid in a Tissue Culture of Berula erecta Grown In Vitro

Chlorogenic acid is produced mainly from extracts of medicinal plants, but the production of chlorogenic acid is usually limited due to the low content in plants and the longer growth cycle [62]. Producing a valuable secondary product in an in vitro culture rather than in the whole crop has several key advantages. These advantages are: production can be more reliable, simple and more predictable; isolation of the phytochemical can be rapid and efficient, as compared to extraction from complex whole plants; compounds produced in vitro can be compared directly to compounds in the whole plant; interfering compounds that occur in the field-grown plant can be avoided in in vitro culture; in vitro cultures can yield a source of defined standard phytochemicals in larger volumes and in vitro cultures are potential models to test elicitation [98]. For the efficient production of secondary metabolites by in vitro culture, it is necessary to optimise the accumulation of biomass of the in vitro culture and the biosynthesis of the selected metabolite [99]. In this study, the highest yield of chlorogenic acid per litre of prepared MS medium was determined in a culture grown at 12 °C under blue light for 28 days. The yield of chlorogenic acid was significantly higher under these conditions than with other treatments, indicating that the above-mentioned combined elicitation is the most suitable for obtaining chlorogenic acid from tissue cultures of *Berula erecta*. The yield of chlorogenic acid in the cell culture of *Cecropia obtusifolia* was lower (15.94 mg/L) than in *Berula erecta* [100]. However, hairy root cultures of *Stevia rebaudiana* have a higher yield of chlorogenic acid than *Berula erecta* [101]. Also, the most efficient hairy root culture of *Leonurus sibiricus* had a higher yield (305.77 mg/L) of chlorogenic acid than that of *Berula erecta* [102]. The peak yield of chlorogenic acid in an in vitro culture of *Gardenia jasminoides* was also higher (232.32 mg/L) than in *Berula erecta* [57]. By comparing the yield of chlorogenic acid of other plant species, it can be concluded that the yield of chlorogenic acid was higher compared to certain plant species and also lower compared to other plant species. For a higher yield of chlorogenic acid it would be necessary to study the biomass accumulation of the in vitro culture of *Berula erecta* and the biosynthesis of chlorogenic acid separately [103]. In such a study, it would be necessary to test the effects of individual elicitors instead of combined elicitation, which would provide a better insight into the physiological response of *Berula erecta* to individual elicitors. Also, other types of elicitation should be tested in the future [55,56,57,58,59,60], and the extraction process of chlorogenic acid from plant material should be optimised [104]. However, obtaining secondary metabolites using the technique of in vitro plant cultures also has disadvantages, such as low biomass yield, higher production costs, the maintenance of a controlled environment, the need for specialised manpower, a small number of profitable large-scale plants and the high cost and complexity of extraction, purification and analysis of the produced metabolites [105]. Consequently, chlorogenic acid can also be obtained by other techniques, such as fungal culture [106], bacterial cultivation [107] and fermentation of coffee pulp [108,109]. The most effective treatment of the fungus *Saccharomyces cerevisiae* studied has a higher yield of chlorogenic acid (234.8 mg/L) than *Berula erecta* culture grown under the most effective combined elicitation in the present study [106]. The most effective strain of bacterium *Escherichia coli* tested also had a much higher yield of chlorogenic acid (472.0 mg/L) compared to the tissue culture of *Berula erecta* [107]. The yield of chlorogenic acid obtained by fermentation of coffee pulp in the da Silveira study was much lower (15 mg/L, after 8 h of coffee pulp fermentation with yeast A) than the yield of chlorogenic acid in *Berula erecta* in the present study [108]. However, the yield of chlorogenic acid during the fermentation of coffee pulp in Myo’s study was higher than the yield of *Berula erecta* throughout the experiment, although it decreased with the duration of the fermentation of coffee pulp [109]. Coffee beans are an excellent source of chlorogenic acid, as well as other phenolic compounds and antioxidants [110,111]. Ethanol extracts of differently roasted seeds contain different concentrations of chlorogenic acid, which are all higher than those of *Berula erecta* [112]. The ethanol extract of coffee beans also contained higher TPC (36.92 mg GA/g DW) [113] and showed higher antioxidant activity (0.572–0.785 mmol Trolox/g DW) [114] than the ethanolic extracts of *Berula erecta*. However, from the comparison of the chlorogenic acid yields obtained with different techniques, we can conclude that the biomass yield and chlorogenic acid yield in *Berula erecta* tissue cultures should be optimised further—studies on the separate effects of light and temperature, as well as studies to test the efficacy of other potential elicitors, would be needed in the future.

## 4. Materials and Methods

### 4.1. Chemicals

The sucrose, MS medium and myo-inositol were supplied by Duchefa (Haarlem, The Netherlands). The Difco Bacto agar was purchased from BD Difco (Franklin Lakes, NJ, USA). The HPLC-grade methanol, HPLC-grade ethanol, HPLC-grade acetonitrile, HPLC-grade acetic acid, sodium carbonate (Na_2_CO_3_ > 99%), 2,2-diphenyl-1-picrylhydrazyl (DPPH) reagent and Trolox (6-hydroxy-2,5,7,8-tetramethylchroman-2-carboxylic acid) were obtained from Sigma-Aldrich (St. Louis, MO, USA). Standard compound chlorogenic acid (99%) was purchased from Acros Organics (Geel, Belgium). The Folin–Ciocalteu reagent was supplied by Merck (Darmstadt, Germany). The gallic acid (99%) was obtained from Carlo Erba (Cornaredo, Italy). The ultrapure water (resistance above 18 MΩ cm) used was obtained from a Milli-Q water purification system.

### 4.2. Plant Material

The plant material of *Berula erecta* was obtained from the collection of plant tissue cultures of the Department of Biology, Faculty of Natural Sciences and Mathematics, University of Maribor, Slovenia. It was grown and propagated on a simple Murashige and Skoog (MS) medium without growth regulators [26]. The MS medium, enriched with 0.8% Difco Bacto agar and 3% sucrose, was adjusted to a pH of 5.7 before autoclaving. Two shoots of approximately equal size were placed on the surface of 20 mL MS medium in 100 mL jars, which were then covered with transparent lids. The experiment was repeated on 42 jars containing a total of 84 shoots. The plants were cultivated under controlled conditions at 23 ± 2 °C, a photoperiod of 16 h at 38–50 μmol m^−2^ s ^−1^ (Osram L 58W/77—Fluora) and 50% relative humidity. After two weeks of growth induction under the previously described conditions, the plants were exposed to combined elicitation under different light and temperature conditions. One third of the plant tissue cultures remained under the same conditions, another third of the plant tissue cultures were exposed to 12 °C and blue LED light (maximum emission peak at 480 nm) and one third of the plant tissue cultures were exposed to 12 °C and red LED light (maximum emission peak at 660 nm). The photoperiod and humidity were the same under all the tested conditions. Plant development, fresh weight, dry weight, chlorogenic acid content, total phenolic compound content and antioxidant activity of the extracts were determined for the plant samples. The plant samples were taken immediately at the beginning of the experiment, after 14 days under all three different light and temperature conditions and after 28 days under all three different light and temperature conditions. The experiment, with 6 replicates per treatment, was repeated twice. The results of the two repetitions did not differ significantly from each other, therefore, the results of both replicates are presented together.

### 4.3. Plant Development

Plant development was assessed at the beginning of the experiment and after 14 and 28 days. The visual quality of the plants was assessed based on visible changes (plant size, leaf colour, presence of chlorosis and necrosis).

### 4.4. Determination of Fresh and Dry Weights

Fresh and dry biomass content was measured immediately at the beginning of the experiment, after 14 days under all three different light and temperature conditions and after 28 days under all three different light and temperature conditions. The fresh and dry weights were measured using an analytical balance (Sartorius analytic).

### 4.5. Ultrasonic Extraction (UE) of Bioactive Compounds from Berula erecta

Precisely 5.00 mL of 70% ethanol was added to 250 mg of each lyophilised sample into a conical centrifuge tube. The mixture was sonicated in an ultrasonic bath (Vevor, Shanghai, China) for 30 min at room temperature, and, after that, centrifuged for 10 min at 10,000 rpm at room temperature (Centrifuge Model 5804 R, Eppendorf, Hamburg, Germany). The supernatant was transferred into a 10 mL measuring flask. After that, another 5 mL of 70% ethanol was pipetted onto the pellet in a conical centrifuge tube. The sonication and centrifugation were repeated under exactly the same conditions as previously. The supernatant after the second centrifugation was added to the same flask as before, and the flasks were filled up to the mark (10 mL) with 70% ethanol. Afterwards, all samples were filtered through 0.45 μm PTFE filters and transferred into vials prior to further analysis. The extraction was carried out in duplicate. Both parallels were always analysed in the subsequent spectrophotometric and HPLC analyses. The extracts were stored in a dark place at −20 °C until further analysis.

### 4.6. Determination of the Total Phenolic Content (TPC)

The total phenolic compounds were determined spectrophotometrically, according to the standard Folin–Ciocalteu method. For these analyzes, 40 μL of each ethanolic extract (base or diluted extract) was taken and mixed with 3.160 mL of ultrapure water and 200 μL of 10% Folin–Ciocalteu (FeC) reagent in microtubes. After precisely 7 min, 600 μL of Na_2_CO_3_ solution (200 g L^−1^) was added, vortexed and incubated for 2 h at room temperature in the dark. The concentration of total phenolics was analysed spectrophotometrically (Varian Cary 50 Bio spectrophotometer, Varian, Palo Alto, CA, USA) at 765 nm against a blank. The total phenolics were calculated as gallic acid equivalents using the regression equation between the gallic acid standards (50–500 mg L^−1^ gallic acid in pure ethanol). All the samples were analysed in duplicate.

### 4.7. DPPH-Radical-Scavenging Activity of Antioxidants

The antioxidant activity of the extracts was determined using the standard DPPH spectrophotometric method. First, 0.05 mL of each ethanolic extract was mixed with 4.95 mL of DPPH reagent in microtubes. Afterwards, the samples were vortexed and incubated for 30 min at room temperature in the dark. The antioxidant activity of the standards and the samples was measured spectrophotometrically (Varian Cary 50 Bio spectrophotometer, Varian) at 517 nm against a blank. The antioxidant activity of the extracts and standards was calculated as millimoles Trolox (6-hydroxy-2,5,7,8-tetramethylchroman-2-carboxylic acid) equivalents per gram dry weight of plant material (mmol Trolox g^−1^ DW), using the regression equation between standard solutions of Trolox (0.2–5.0 mmol L^−1^ in pure ethanol). All the samples were analysed in duplicate. 

### 4.8. The Identification and Quantification of Chlorogenic Acid Using the HPLC-UV Technique (Chromatographic System, Conditions and Validation of the Method)

The HPLC-UV method used in this work was described previously by Ivanović et al. [115]. The chlorogenic acid in the plant samples was analysed by the HPLC Varian system, coupled with an autosampler (ProStar 410), a binary solvent pump (ProStar 210) and an UV–Vis detector and a column (Agilent XDB-C18 column, 150 mm × 4.6 mm I.D., 5 μm particle size) and a temperature of 25 °C. Chlorogenic acid was detected at a wavelength of 280 nm. Two mobile phases were used to separate the samples: A: 100% acetonitrile and B: 1% aqueous acetic acid solution, which were mixed according to the following gradient method: 0–1 min 95% (B), 1–18 min 74% (B), 18–28 min 68% (B), 28–40 min 59% (B), 40–40.10 min 95% (B), and the column required 7 min to reach an equilibrium state with its initial conditions. The volume of the injected samples was 10 μL, and the flow rate of the mobile phase was set to 1 mL min^−1^. Each sample vial was analysed twice. The chlorogenic acid content was calculated from the peak areas of the samples and with the corresponding standard curve of chlorogenic acid, which was constructed at eight different concentrations (1 mg L^−1^, 5 mg L^−1^, 10 mg L^−1^, 25 mg L^−1^, 50 mg L^−1^, 100 mg L^−1^, 150 mg L^−1^, 200 mg L^−1^) of chlorogenic acid standard (Acros Organics) prepared in acidified methanol. The chlorogenic acid in the samples was identified based on the retention time of the chlorogenic acid standard. The HPLC-UV chromatogram of an ethanolic plant extract and the HPLC-UV chromatogram of the standard of chlorogenic acid are shown in Figure 3 (Section 3). Chlorogenic acid isomerises rapidly from *trans*- to *cis*-isomer [116,117,118]. Therefore, it was detected with two peaks in the HPLC-UV chromatogram. The peak areas of *trans*- and *cis*-isomer were summed for correct evaluation of the result. The retention time of the *trans*-chlorogenic acid was 9.98 min, and the retention time of the *cis*-chlorogenic acid was 12.47 min. In this study, the method was validated for linearity, intra-day precision, limit of detection (LOD) and limit of quantification (LOQ). The linearity of the curve was confirmed by a linear least squares regression (R^2^ = 0.9997) and the quality coefficient (QC = 2.45%). The intra-day precision was expressed as relative standard deviation, which was 0.4%. The LOD and LOQ for chlorogenic acid in this study were 1.61 mg L^−1^ and 5.35 mg L^−1^, respectively. The content of chlorogenic acid in the samples was expressed in mg/1 g DW. The yield of chlorogenic acid was calculated based on the concentrations of chlorogenic acid and dry weight that can be obtained in one litre of MS medium. The yield of chlorogenic acid was expressed as mg of chlorogenic acid/1 L MS medium.

### 4.9. Statistics

The plant biomass results, which were calculated from two duplicates, are represented by mean values (n = 24) and standard deviations (±SD) and were evaluated statistically by one-way analysis of variance (ANOVA) using the SPSS 21 software (SPSS Inc., Chicago, IL, USA). Significant differences between the mean values were determined using the Kruskal–Wallis test followed by Dunn’s post hoc test. Significant differences (*p* < 0.05) between the mean values were indicated by different letters. The results of the biochemical analyses (chlorogenic acid content, TPC, antioxidant activity, yield of chlorogenic acid) are given as mean values (±standard error, SE) of the analyses on plant material cultivated in two replicates. The measurements were performed at least twice for each sample and in duplicate. The normality assumptions for all the biochemical traits were checked using the Kolmogorov–Smirnov test. In addition, one-way analysis of variance (ANOVA) was used to test for differences between the combined effect of elicitation and biochemical traits (SPSS Inc., Chicago, IL, USA). The post hoc Duncan test was employed for biochemical traits that were evaluated as significant (*p* < 0.05). The Pearson correlation coefficient (sig. 2-tailed) was calculated between the TPC and antioxidant activity with respect to the chlorogenic acid content.

## 5. Conclusions

The potential of the plant species *Berula erecta* for the production of chlorogenic acid and the influence of combined elicitation with light and temperature on this were investigated in this study. It was found that the tissue culture of *Berula erecta* reached the highest fresh and dry weights when it was cultivated at 23 °C and under white light. The plants that were cultivated for the longest time also achieved a higher weight compared to the plants that were cultivated for a shorter time. When the plants were exposed to 12 °C and blue or red light, the increase in plant biomass slowed down. This is the first study in which chlorogenic acid has been identified and quantified in *Berula erecta*. The plant samples grown under all the tested conditions contained chlorogenic acid. Plants grown for 28 days after the beginning of the experiment at 12 °C and under blue light had the highest chlorogenic acid content. The same applies to the total phenolic compound content and the antioxidant activity of the ethanolic extracts. It can be concluded that this was the result of the plant’s response to exposure to abiotic stress factors. Compared to some other plant species grown in vitro, *Berula erecta* shows potential for in vitro production of chlorogenic acid. Many more studies would need to be conducted before *Berula erecta* could be used for the production of chlorogenic acid. In particular, it would be necessary to test the influence of lower temperature and light of different wavelengths separately—with these findings on the physiological response of *Berula erecta*, the combined elicitation could be optimised more effectively in future studies, and, in particular, investigations to optimise the triggering of the production of chlorogenic acid further and also on the identification of other bioactive compounds in *Berula erecta* should be performed in the future. In addition, the production of chlorogenic acid in the tissue culture of *Berula erecta* would need to be researched on a large scale.

## Figures and Tables

**Table 1:** Mean content (±SE) of chlorogenic acid and total phenolic in *Berula erecta* grown in vitro (n = 24) in relation to different light and temperature conditions. The different letters (a–g) indicate significant differences (*p* < 0.05), which were determined using a Kruskal–Wallis test.

Treatment	Content of Chlorogenic Acid [mg/1 g DW]	Total Phenolic Content [mg GA/1 g DW]
The beginning of the experiment	1.893 ± 0.020 e	17.29 ± 0.11 e
14 days, white light, 23 °C	1.309 ± 0.002 f	16.41 ± 0.35 f
28 days, white light, 23 °C	3.042 ± 0.019 b	23.77 ± 0.66 b
14 days, blue light, 12 °C	2.604 ± 0.022 c	21.30 ± 0.24 c
28 days, blue light, 12 °C	4.049 ± 0.021 a	28.12 ± 0.38 a
14 days, red light, 12 °C	0.927 ± 0.014 g	14.76 ± 0.28 g
28 days, red light, 12 °C	2.050 ± 0.012 d	20.30 ± 0.13 d

**Table 2:** Mean antioxidant activity of ethanolic extracts (±SE) of *Berula erecta* grown in vitro (n = 24) in relation to different light and temperature conditions. The different letters (a–e) indicate significant differences (*p* < 0.05), which were determined using the Kruskal–Wallis test.

Treatment	Antioxidant Activity [mmol Trolox/1 g DW]
The beginning of the experiment	0.0485 ± 0.0002 d
14 days, white light, 23 °C	0.0469 ± 0.0012 d
28 days, white light, 23 °C	0.0687 ± 0.0006 b
14 days, blue light, 12 °C	0.0649 ± 0.0016 bc
28 days, blue light, 12 °C	0.1008 ± 0.0003 a
14 days, red light, 12 °C	0.0296 ± 0.0007 e
28 days, red light, 12 °C	0.0638 ± 0.0013 c

**Table 3:** Pearson’s correlation coefficient between TPC and antioxidant activity in respect to chlorogenic acid content. ** Correlation is significant at the 0.01 level (2-tailed).

Spectrophotometric Assay	Chlorogenic Acid Content
TPC	0.980 **
Antioxidant activity (DPPH assay)	0.963 **

**Table 4:** Mean yields of chlorogenic acid (±SE) obtained from *Berula erecta* grown in vitro (n = 24) per 1 L of prepared MS medium in relation to different light and temperature conditions. The different letters (a–g) indicate significant differences (*p* < 0.05), which were determined using the Kruskal–Wallis test.

Treatment	Yield of Chlorogenic Acid Obtained in Tissue Culture of *Berula erecta* per Litre of Prepared MS Medium [mg/1 L MS Medium]
The beginning of the experiment	20.846 ± 0.220 f
14 days, white light, 23 °C	29.787 ± 0.046 d
28 days, white light, 23 °C	126.874 ± 0.792 b
14 days, blue light, 12 °C	22.683 ± 0.192 e
28 days, blue light, 12 °C	168.873 ± 0.265 a
14 days, red light, 12 °C	9.402 ± 0.142 g
28 days, red light, 12 °C	32.520 ± 0.190 c

## Data Availability

Data are contained within the article.
